# COVID-19 outbreak management in Western Sydney residential aged care homes: A mixed-methods Donabedian evaluation

**DOI:** 10.1371/journal.pone.0318490

**Published:** 2025-03-20

**Authors:** Vincent V. Vicencio, Catherine Viengkham, Nicholas Grange, Sophie Norton, Ramon Z. Shaban

**Affiliations:** 1 Population and Community Health, South Eastern Sydney Local Health District, Taren Point, New South Wales, Australia; 2 Sydney Infectious Diseases Institute, Faculty of Health and Medicine, University of Sydney, Taren Point, New South Wales, Australia; 3 Research and Education Network, Western Sydney Local Health District, North Parramatta, New South Wales, Australia; 4 NSW Ministry of Health, Sydney, Australia; 5 New South Wales Biocontainment Centre, NSW High Consequence Infectious Disease Specialist Service, North Parramatta, New South Wales, Australia; 6 Centre for Population Health, Western Sydney Local Health District, New South Wales, Australia; 7 Susan Wakil School of Nursing and Midwifery, Faculty of Health and Medicine, University of Sydney, Taren Point, New South Wales, Australia; City University of New York, UNITED STATES OF AMERICA

## Abstract

Outbreaks of the novel respiratory viral disease, SARS-CoV-2 (COVID-19), have caused disproportionate morbidity and mortality for older people living in residential aged care homes. Between June 2021 and December 2022, the Delta and Omicron variants of COVID-19 were responsible for widespread outbreaks in homes across Western Sydney, New South Wales, Australia. To manage outbreaks in affected homes, a targeted response strategy was prepared and deployed in the form of outbreak management teams. This study utilised the Donabedian framework and a two-phase mixed methods design to evaluate the structures, processes and outcomes of the outbreak management teams at the level of the local health district. Phase 1 involved the descriptive analysis of outbreak data from Western Sydney aged care homes, created between June 2021 and December 2022. Phase 2 involved the completion of in-depth semi-structured interviews with 35 participants to explore the outbreak management team response from the perspective of its members and staff from residential aged care homes. Between June 2021 and December 2022, there were 281 outbreaks, 4113 resident cases, 346 hospitalisations and 127 deaths in residential aged care homes across Western Sydney. Structural factors that facilitated the outbreak management response and improved outcomes included smaller home sizes, the absence of shared rooms and bathrooms, adequate staffing and resources, suitable infrastructure, and the integration of the response with wider public health systems. Process facilitators included multi-disciplinary team membership, open communication channels, structured and streamlined procedures and roles, onsite infection control support and education, and long-term capability building. The lessons drawn from participants’ experiences aim to improve the outcomes and sustainability of current and future outbreak management strategies.

## Introduction

Older individuals, particularly those living in residential aged care homes (RACH), are at higher risk of adverse outcomes from communicable diseases like COVID-19 [1, 2]. Some 75% of all COVID-19 related deaths in Australia in the first year of the pandemic were of individuals in RACHs [3]. This increased risk still persists in the present day where, at time of writing, RACH residents comprise approximately 26% of all directly-attributable COVID-19 deaths in Australia [4, 5].

The risk factors that augment these negative outcomes are multi-faceted and well-established in the literature [1,6–8]. On one hand, older persons are intrinsically more susceptible to both contracting and experiencing more severe complications from communicable diseases due to physiological predispositions associated with ageing, such as frailty and generally weakened immune systems [9–13]. Higher rates of comorbidities and chronic conditions, such as diabetes, heart disease and obesity, also persist in this population and greatly increase the likelihood of severe illness [6,8]. Furthermore, neurological conditions such as dementia, as well as functional disabilities, often inhibit the older person’s ability to adhere to fundamental infection prevention and control (IPC) measures [14–16]. These intrinsic risks are further compounded within the environment of a RACH, a service that provides a home-like communal setting where residents may live on a permanent or respite basis. In RACHs, the common use of shared living spaces and homes, close proximity and frequent interactions with transient staff and guests, all increase the risk of outbreaks and disease spread [17]. Furthermore, while most homes have the capacity to provide clinical care through a multidisciplinary workforce that includes registered nurses and allied health professionals, they are often significantly inhibited by infrastructural limitations, lack of access to clinical resources and high workloads due to inconsistent staffing [17–19]. The arrival of the Delta variant of COVID-19 in June 2021 saw an exponential increase in outbreaks in RACHs across Australia’s most populous state, New South Wales (NSW) [20, 21]. In NSW, the management of public hospitals and healthcare facilities are the responsibility of their delegated local health districts (LHD). LHDs are operated by the state government health department (NSW Health), and work in tandem with a number of specialty networks, primary health networks and other public health organisations to provide healthcare services within a defined geographical area. The state of NSW is divided 15 LHDs, including six metropolitan LHDs that cover the Sydney metropolitan region, and nine regional and rural LHDs that encompass the remainder of the state.

In response to the pandemic, NSW Health released an *Incident Action Plan* [22] in May 2020, which followed the advice of contemporaneous federal guidelines [23] and detailed the state’s public health response to confirmed cases of COVID-19 in RACHs. Core to the response was the establishment of an outbreak management team (OMT) for every identified COVID-19 outbreak in a RACH. The OMT was responsible for assisting RACHs with the completion of their outbreak management plan (OMP), coordinating IPC measures, assessing staff and IPC resource, and identifying and managing all COVID-19 cases in the facility, including initiating appropriate actions for contact tracing and resident welfare [22,24,25]. Membership on the OMT was purposefully diverse, multi-disciplinary and adaptive to developing needs over time. At minimum, the team needed to include a manager, or equivalent representative, from the affected RACH, a delegate from the LHD’s Public Health Unit (PHU) and the Public Health Emergency Operations Centre (PHEOC), a clinical representative and an IPC specialist [22,26]. The OMT formed part of a systemic response, with a clearly articulated structure for escalation that engaged multiple levels of local, state and federal health authorities to facilitate an all-systems approach [26].

The OMT response was uniquely characterised by its rapid and targeted approach. The assembly of the OMT and the scheduling of the first meeting occurred within 12 hours of the outbreak notification from the affected home [22]. The OMT operated as the key point of contact between the RACH and the state’s various public health structures and resources. The first OMT meeting would be the initial point of information transfer from the RACH to the OMT, facilitating an assessment of needs that would inform the subsequent actions that needed to be undertaken. These actions would include, for example, site visits to provide on-the-ground IPC support, training and education, and the provision of PPE and testing equipment. Meetings were scheduled to occur daily to monitor progress until the outbreak in the home was resolved. Broadly speaking, the aims of the OMT were to reduce morbidity and mortality within homes, as well as the demand for acute care via emergency departments by mitigating the impact of the outbreaks [27].

Internationally, the OMT response has been observed to be effective in reducing the incidence and burden of COVID-19, with significant support and acceptance by RACH management and staff [28, 29]. However, the pace of the pandemic which necessitated continual and rapid changes to outbreak practices have been associated with decreased wellbeing in RACH staff [30, 31]. Domestically, there is a paucity in evaluations of responses to COVID-19 outbreaks in RACHs, particularly in NSW. Some studies from other Australian states have reported on the outcomes of RACH outbreaks in relation to home, resident and geo-temporal risk factors [32, 33]. In NSW there are some published accounts of single home outbreaks, but a lack of literature at the LHD level which had the direct responsibility for COVID-19 outbreak management [34, 35].

This paper specifically examines the OMT response of the Western Sydney Local Health District (WSLHD), which is one of the six metropolitan LHDs, covering an area of 780 km^2^ to the west of Sydney’s central business district, with an estimated population of nearly one million people. The WSLHD response is significant as the Western Sydney area became one of the major epicentres for COVID-19 transmission during 2021 Delta-variant wave, both in the community and in RACHs [36]. Given the fundamental importance of and formal legislative requirements for OMTs in these settings, this study aimed to explore and characterise the structures, processes and outcomes of OMTs for the prevention and management of COVID-19 outbreaks in Western Sydney RACHs.

## Methods

### Study design

**Fig 1:**
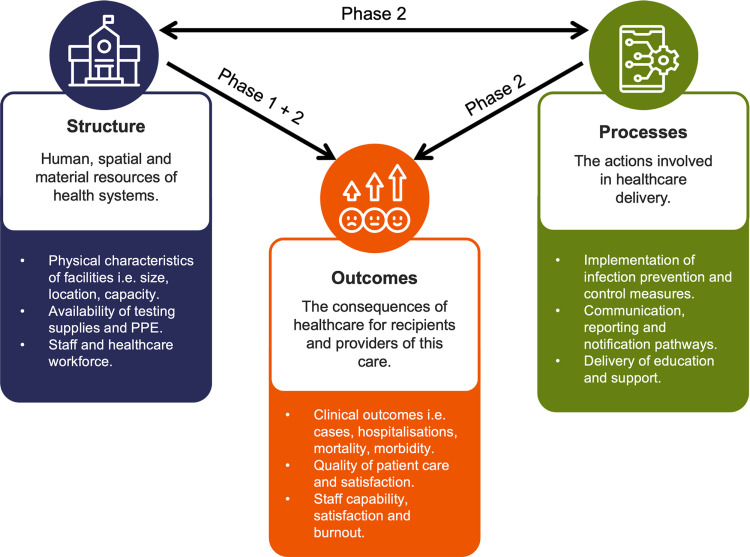
This study used the Donabedian framework to explore the interdependent components of structure, processes and outcomes of the OMT response. Phase 1 examined the relationship between structures and outcomes. Phase 2 examined both the relationship between structures and processes, as well as between processes and outcomes.

This study was guided by the Donabedian framework for the evaluation of health systems (Fig 1) [37]. Structure pertains to the human, spatial and material resources of health systems. Processes pertain to the actions involved in healthcare delivery. Outcomes are the consequences of healthcare for recipients and providers.

This study adopted an explanatory sequential mixed methods design [38]. Phase 1 involved the collation of data collected from state-base surveillance systems, surveys and document analysis to inform the structure and outcomes of the response. This data then informed Phase 2, which employed semi-structured interviews with members of OMTs and RACHs to further characterise the relationship between structures and processes, as well as between the processes and outcomes of the OMT response. The qualitative data obtained in Phase 2 was prioritised in the interpretation of findings as it allowed for the interrogation of aspects of the Donabedian framework (i.e., Structure ↔  Processes, Processes →  Outcomes) that could not otherwise be examined using the quantitative data alone.

Human research ethics and governance approval was obtained from the WSLHD Human Research and Ethics Committee (2022/ETH01938), and the study was conducted in accordance with the approved protocol. A combination of written and verbal consent was obtained from all participants in this study.

### Study population and participants

All RACHs that were operating within WSLHD and that experienced an outbreak between June 2021 to December 2022 were included in this study. This period spans the pre-eminence of the Delta (B.1.617.2) and Omicron (B.1.1.529) variants of concern until the abolition of COVID-19 Public Health Orders in NSW [33]. All WSLHD RACHs and current and former WSLHD staff involved in COVID-19 OMTs during this period were eligible and invited to participate. Contact details were obtained through an existing database maintained by the public health unit (PHU) as part of outbreak management operations. WSLHD staff were purposively sampled based on their role in the OMT (e.g., PHU delegate, clinician, IPC specialist, executive) during the relevant period, ensuring that at least one participant from each role was included. RACH staff were convenience sampled from the database. Data for Phase 1 were collected from a total of 64 RACHs. Data for Phase 2 were collected from 35 interview participants, including 27 were staff from WSLHD and 8 from RACH management (representing 7 out of 63 eligible RACHs in WSLHD).

### Data collection and analysis

#### Phase 1.

Phase 1 collated the data generated from RACHs and the outbreak management process during the analysis period. Data was primarily drawn from four sources: the NSW Notifiable Conditions Information Management System (NCIMS), a database maintained by the WSLHD PHU containing information relevant to RACH characteristics, documents generated during OMT correspondence, and the Australian Institute of Health and Welfare’s (AIHW) GEN Aged Care Data [39]. All relevant databases were accessed by the research team on the 15^th^ of March 2023. No individually identifiable information was accessed or collected in this phase.

NCIMS is a confidential application operated by the NSW Public Health Network (PHN) to capture and maintain state-wide data of medical conditions notifiable from pathology laboratories, general practitioners and hospitals. The following variables were extracted from NCIMS for each outbreak event: home identity, location, type, date of outbreak notification, outbreak length – and for both staff and residents – the date of onset of the first and last cases, the number of laboratory-confirmed cases, the number of hospitalisations and the number of deaths. Where NCIMS data was incomplete it was supplemented through a review of contemporaneous correspondence and associated documents generated via interaction with WSLHD staff and the home. These included OMT meeting minutes, transcripts, email correspondence and outbreak progress logs [40].

Additionally, data pertaining to various structural and organisational characteristics of individual RACHs were obtained from a database maintained by the WSLHD PHU as part of outbreak management operations. Information in this database were collected from homes prior to the current study and included information such as the home’s ability to cohort residents, number of floors, and the number of distinct sections in the home, including the presence of dementia-specific sections. This data was supplemented by publicly available data from GEN Aged Care, which included the number of residential places, organisation type, number of additional homes administered in NSW by the provider, and whether the home had shared bedrooms and/or bathrooms [39].

#### Phase 2.

Phase 2 data was collected through in-depth, semi-structured interviews with members of the WSLHD and RACH OMTs. Interviews were conducted by five members of the research team. All interviewers had experience in either outbreak management or public health, but none had direct associations with members of the WSLHD OMTs during the analysis period. Two interview guides were developed – one for OMT members and one for RACH members – and piloted between interviewers to ensure consistency. Both interview guides featured open-ended questions, which were divided into three sections corresponding to the Donabedian framework. The OMT interview guide contained 30 questions, and the RACH guide contained 40 questions. The guides were developed based on prior knowledge of, and experience with OMTs, as well as from the outcomes of Phase 1.

Invitations to participate in the interviews were distributed to participants via email. Informed consent was obtained in both written form and verbally. Participant information packages were distributed with interview invitations, which contained an information statement and consent form. Participants were asked to sign and return the consent forms via email prior to their interviews. If written consent had not been received prior to the interview, the interviewer provided a verbal overview of the information and consent statement and requested verbal consent from the participant before commencing the interview. Verbal consent was recorded and saved separately from the interview transcript. Recruitment began on the 29^th^ of June 2023 and closed on the 15^th^ of September 2023.

All interviews began with a review of the outbreak timeline where participants were provided with a document that charted and summarised COVID-19 case numbers and deaths in WSLHD RACHs during the analysis period. For RACH members, individualised documents were distributed that also included case numbers and outbreaks for the home in which the participant was employed during the analysis period. Both the outbreak document and a summary of the interview questions were distributed to participants via email one day prior to their scheduled interviews. Interviews were approximately 60 minutes in length. All interviews were completed in Microsoft Teams (Microsoft Corporation, Redmond, WA, USA) and recorded for transcription and analysis. Audio recordings were used to assist in the cleaning of interview transcripts and were deleted after the transcription process was completed. Identifiable information was redacted from transcripts and references to specific information (e.g., organisation names) were replaced with broader jurisdictional descriptors. All interview and administrative data are stored securely in the Research Data Store (RDS) maintained by the University of Sydney.

Data collected in Phase 2 were analysed using Braun and Clarke’s method for qualitative thematic analysis [41]. Anonymised transcripts were imported into NVivo 14 (Lumivero, Denver, CO, USA) for management and coding. Initial codes were generated a priori from the structure of the interview guide. Themes and sub-themes were then independently extracted and categorised by two coders (VVV, CV) following further thematic analysis. Rigour was ensured through crosschecking and agreeing on generated themes by the two coders, before these themes were member checked.

## Results

Data from Phase 1 and Phase 2 were examined together to explore impact of structural factors on the outcomes of the OMT response, specifically the number of resident cases, hospitalisations and deaths following outbreaks. Phase 2 data further explored the relationship between structural and process factors, as well as the effect of process factors on outcomes pertaining to the members of the RACH and OMT workforce, particularly with regards to their wellbeing, capability and knowledge.

### Structure and outcomes

The characteristics of WSLHD RACHs during the analysis period are summarised in Table 1. There was a total of 63 RACHs operating in WSLHD in 2021 and 64 in 2022. Over 40% of RACHs identified as private incorporated bodies and almost half (46 - 46.9%) belonged to larger organisations that administered more than 11 homes in NSW. In terms of infrastructure, between 69.8 – 71.9% of RACH indicated a mix of single and shared rooms. Almost all (98.4%) indicated having 2 or more distinct sections in the home and between 84.1 – 84.4% indicated they had the ability to cohort residents.

**Table 1:** Characteristics of WSLHD RACHs, by year of operation as of 1 July. Ability to cohort residents, dementia specific sections, number of floors and distinct sections are all self-reported from homes. Number of homes administered refers to the number of additional sites operates by the same provider in NSW.

Characteristic	Year	
**2021 (n = 63)**	**2022 (n = 64)**
*Residential places, mean (SD)*	102.4 (40.9)	101.5 (41.6)
*Total residential places, n (%)*		
<40	2 (3.2)	3 (4.7)
40-79	18 (28.6)	17 (26.6)
80-119	19 (30.2)	20 (31.2)
120-159	20 (31.7)	20 (31.2)
160 +	4 (6.3)	4 (6.2)
*Organisation type, n (%)*		
Charitable	11 (17.5)	12 (18.8)
Community Based	4 (6.3)	4 (6.2)
Private Incorporated Body	28 (44.4)	28 (43. 8)
Religious	20 (31.7)	20 (31.2)
*Number of homes administered n (%)*		
1	18 (28.6)	18 (28.1)
2-5	11 (17.5)	11 (17.2)
6-10	5 (7.9)	5 (7.8)
11 +	29 (46.0)	30 (46.9)
*Room types in home, n (%)*		
Mix of single and shared rooms	44 (69.8)	46 (71.9)
Single rooms only, no shared bathrooms	19 (30.2)	18 (28.1)
*Dementia specific places, mean (SD)*	15.1 (15.3)	15.1 (15.3)
*Distinct sections in home, mean (SD)*	4.3 (2)	4.3 (2)
*Number of distinct sections in home, n (%)*		
1	1 (1.6)	1 (1.6)
2-4	40 (63.5)	40 (62.5)
5-7	16 (25.4)	17 (26.6)
8 +	6 (9.5)	6 (9.4)
*Number of floors in home, n (%)*		
1	17 (27.0)	17 (26.6)
2 +	46 (73.0)	47 (73.4)
*Ability to cohort residents, n (%)*		
Yes	53 (84.1)	54 (84.4)
No	10 (15.9)	10 (15.6)

In July 2021, there were 5580 residents living in WSLHD RACHs [39]. Approximately 64.8% of residents were women, which closely corresponds to the national proportion of 66% [42]. Age also reflected national distributions, whereby 97.6% of residents were aged 65 years or over, and 55.7% were aged 85 years or over. The majority of WSLHD residents were born in Australia (50.5%), spoke English as their preferred language (77.2%) and were non-Indigenous (98.4%).

**Fig 2:**
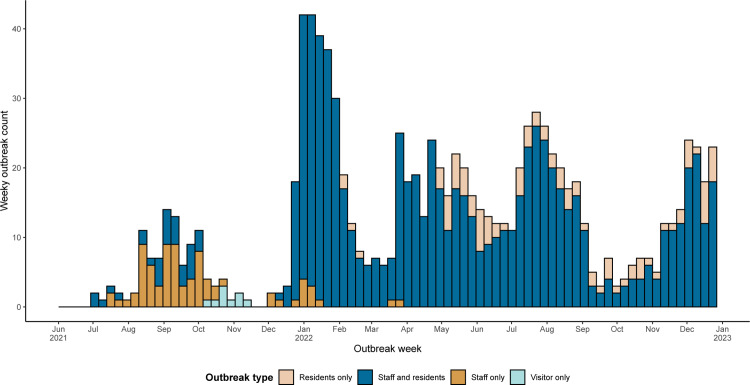
Timeline of WSLHD RACH notified outbreaks, by outbreak type between June 2021 and December 2022. Each count on the y-axis represents a week in which a home spends at least one day in outbreak. Outbreaks which continue across multiple weeks are represented multiple times along the x-axis. Visitor outbreaks represent declared home outbreak where a COVID-19 positive visitor attended the home, an outbreak was declared, with no subsequent staff or resident cases.

**Fig 3:**
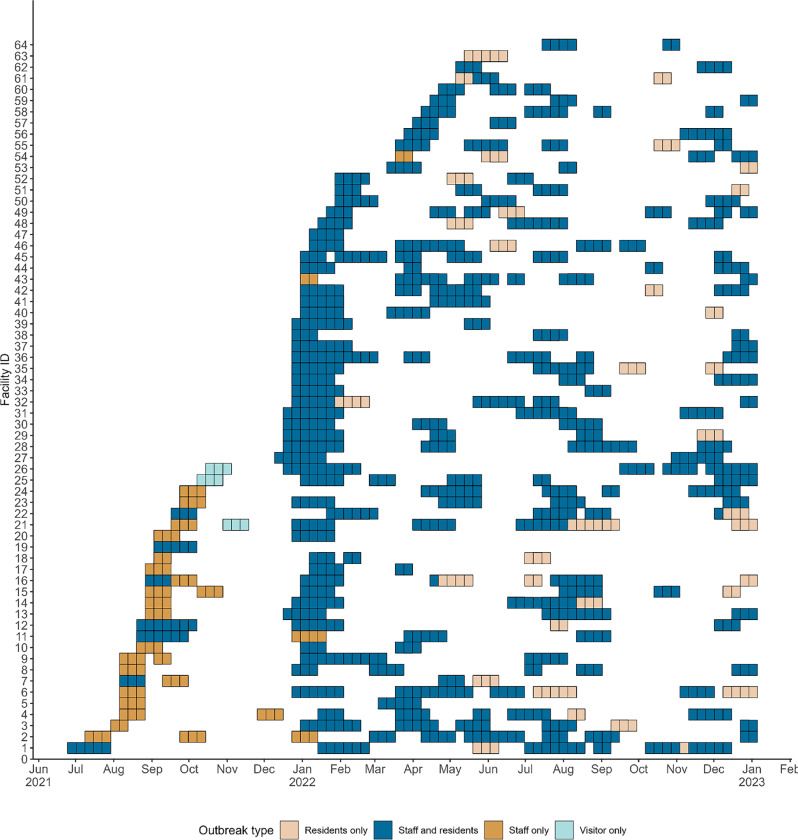
Timeline of outbreaks in WSLHD, by type of outbreak. Each cell represents a week in which the home was in outbreak for at least one day. Each value on the y-axis represents a single home in WSLHD.

During the Delta period, there were 52 outbreaks in WSLHD RACHs, resulting in 621 resident cases, 98 hospitalisations and 48 deaths; as well as 724 staff cases. The Delta period saw a higher number of staff only outbreaks compared to Omicron, which commensurately resulted in a higher number of staff cases compared to residents. The average length of outbreaks during the Delta period was 22.6 days. During the Omicron period, there were 229 outbreaks in WSLHD RACHs, resulting in 3492 resident cases, 248 hospitalisations and 127 deaths. Staff accounted for a smaller proportion of total cases compared to the Delta period, with a total of 1481 staff cases. This period also saw the highest peak in cases during the analysis period in January 2022, which coincided with the lifting of public health restrictions following the systematic rollout of the vaccinations, in addition to rise of the Omicron variant. The average length of outbreaks during the Omicron period was 18.4 days. Fig 2 provides a timeline of RACH outbreaks in WSLHD during the period of interest and Fig 3 shows the distribution of outbreaks over time, as well as their length, for each WSLHD home.

### Impact of home layout and structural characteristics

Resident incidence, hospitalisation and death rates per 1000 person years at risk disaggregated by home characteristics were also determined (Table 2). In general, rates of resident incidence, hospitalisations and deaths were higher during the Omicron period compared to the Delta period. Factors such as the ability to cohort residents and the absence of dementia-specific sections were associated with lower rates across all three outcomes. Larger homes, such as those with greater bed numbers, or multiple floors or distinct sections were also associated with greater incidence, hospitalisation and death rates across both Delta and Omicron periods. Homes that belonged to larger organisations that administered multiple facilities also exhibited lower outbreak rates compared to single-standing homes.

**Table 2:** Rate of resident cases, hospitalisations and death in Western Sydney aged care residents, outbreak period and home characteristics, per 1,000 person years at risk. For the Delta period, time at risk was calculated from the first case of local transmission in NSW – 16 June 2021. For the Omicron period, time at risk was calculated from 1 January 2022 to 31 December 2022.

	Delta period			Omicron period			Overall		
Characteristic	Cases	Hosp.	Deaths	Cases	Hosp.	Deaths	Cases	Hosp.	Deaths
*Total residential places*									
<40	649.1	155.8	51.9	428.6	19	19	487.8	55.7	27.9
40-79	161	15.9	14.2	457.4	24.2	8.4	346.9	21.1	10.6
80-119	162.3	44.2	10.6	542.8	53.4	19.3	413.5	50.3	16.3
120-159	190.7	17.3	15.3	551.7	35.7	24.4	424.7	29.3	21.2
160+	148.4	29.2	4.9	589.7	26.4	17.2	434.5	27.4	12.8
*Organisation type*									
Charitable	28.2	1.8	3.5	468.2	20	19.1	322.9	14.0	14.0
Community Based	362.9	33.9	14.5	566.9	65.6	34.1	495.2	54.5	27.2
Private Incorporated Body	235	41.1	19.9	591.2	37.9	21.4	465.5	39.0	20.9
Religious	131.9	20.6	6.5	488.6	44	14	361.4	35.7	11.3
*Number of homes administered, n (%)*									
1	208.2	52.8	17.6	593.9	42.8	17.7	457.5	46.3	17.6
2-5	137.4	10.3	10.3	543.8	30.7	14.9	401.0	23.5	13.3
6-10	234.7	38	13.8	473.8	43.1	13.1	389.7	41.3	13.4
11+	162.1	16.8	10.6	511.8	37.1	23.5	390.5	30.1	19.0
*Room types in home*									
Mix of single and shared rooms	218	34.4	15.7	542.9	39.2	21.4	431.3	37.6	19.4
Single rooms only, no shared bathrooms	57.7	9.1	4.5	519.7	34.7	13.4	348.2	25.2	10.1
*Dementia specific section*									
Yes	177.3	28.4	12.3	548.7	40.5	20.2	432.0	37.4	19.6
No	178.9	24.8	17.4	451.5	20.2	14.8	353.5	26.8	10.4
*Number of distinct sections in home*									
1	0	0	0	235.3	19.6	19.6	152.5	12.7	12.7
2-4	136.8	31.8	11.1	538.1	35.3	18.9	395.5	34.1	16.1
5-7	212.4	15	17.9	525.3	41.5	20.5	419.1	32.5	19.6
8+	281.9	41.1	10.3	579.2	43.5	20.1	474.7	42.7	16.6
*Number of floors in home*									
1	133.7	11.8	9.5	471.3	28.7	15.3	349.7	22.6	13.2
2+	191.5	33.2	13.9	557.4	41	20.8	430.5	38.3	18.4
Overall	177.5	28	12.9	537.6	38.2	19.6	411.5	34.6	17.2

These finding were supported by data from Phase 2, where several physical and functional characteristics of homes were directly identified by participants as barriers to the efficacy of OMTs. Home layout became one of the primary factors that OMTs sought to ascertain during initial OMT meetings due to its substantial impact on how IPC processes could be effectively applied. When asked to describe the characteristics of homes which made infection control difficult, 71.4% of participants identified home layout as a key challenge during the outbreak response. Homes that were large or multi-storey, as well as those that had many shared and communal spaces, were noted as being particularly problematic for the purposes of zoning and implementing infection control measures.

…we do have… shared rooms here as well and… that was one of our first outbreaks which… the transmission in that concessional shared area just went straight through, straight, straight through the home. (RACHP001)

…older facilities, those with shared rooms and… common bathrooms… just more densely populated facilities in general made life more complicated in terms of maintaining infection control. (OMTP015)

Contrary to Phase 1 findings, several participants described the benefit of having distinct sections or ‘wards’ in homes as these layouts facilitated zoning and cohorting practices. In some cases, RACH managers were able to fully isolate select sections when an outbreak occurred.

…we’ve got 7 different areas, wards if you like, which lent themselves really nicely to be able to be zoned very, very easily., So… it’s very easy for us to lock down the zone if you like… (RACHP001)

Our second last… outbreak was in memory support and goes without saying it’s impossible to isolate… we just closed the whole unit. We’re lucky that it’s separate and has a separate entrance, and we know now how to manage the catering and cleaning and how to bring in and take out… (RACHP002)

However, issues with zoning and isolation still persisted in homes with distinct sections, often due to complex layouts, lack of separate entrances or exits, shared connecting passages or communal spaces, and a lack of built-in infrastructure that effectively restricted access from one section to another.

But if the infection was in [Wing 1 Name], which is the furthest away, they still have to… walk past [Wing 2 Name]… (RACHP002)

…the outbreak started in Wing 3 …but there’s no actual lock. Like you can close the door… there’s a thing you can twist… but if you untwist… the door will open. (RACHP007)

Additionally, 68.6% of participants acknowledged the challenge of homes with residents with dementia and cognitive impairments. In particular, homes with residents that exhibited wandering behaviours were found to place significant strain on the home’s workforce, capacity and resources. Residents with these characteristics typically presented with more severe clinical needs and also with greater non-compliance and resistance to infection control measures, such as cohorting, social distancing and general hygiene practices. As the total number of outbreaks grew during the Omicron period, increased rates of hospitalisation and deaths were observed in homes with dementia-specific units, an observation which this participant shared:

… everyone is gonna get COVID in that dementia specific home because they all are … wandering around. So the infection control measures were very, very hard to implement … (OMTP018)

### Structures and processes

Phase 2 explored the relationship between structural and process factors, including how each factor facilitated or inhibited the other during the OMT response. Key findings included the integration of the OMT within the wider public health response, resources and staffing within the OMT and in the RACH, and the availability and accessibility of physical and digital infrastructure within the OMT workspace. The advantages and disadvantages of various structural and process factors are summarised in Table 3.

**Table 3:** Overview of the structural and process factors that produced positive and negative outcomes associated with the OMT response, as guided by the Donabedian framework.

	Theme	Facilitators/Advantages	Barriers/Disadvantages
**Structures**	**Home Layout & Characteristics**	Homes with layouts that allowed for effective sectioning and cohorting.	Larger home size. Complex home layouts with multiple floors or sections. Presence of dementia patients, dementia-specific units and/or memory-support units. Presence of shared rooms and bathrooms.
	**Resources & Staffing**	Homes administered by a larger organisation to enable the sharing the resources and staff. Secondment of healthcare staff to support administrative roles within the OMT. Establishing supply chains to meet the resource needs of RACHs.	Inadequate access to PPE and testing supplies. Poor storage capacity leading to inability to properly store resources. Staff shortages within RACHs caused by outbreaks and furloughing, as well as within the OMT after seconded staff return to their substantive roles. Poor initial training, education and preparation in staff prior to the start of an outbreak.
	**System Level Integration**	Integration of OMT response within local, state and national bodies. Multi-level organisational engagement and partnership.	Over-involvement can be resource-intensive and potentially excessive if purpose is not well-defined.
	**Physical Workspaces & Digital Infrastructure**	Consistent access to physical workspaces (i.e., desks, computers, phones) and digital infrastructure (i.e., communication software) by members of the public health team. Established and dedicated channels for communication (e.g., MS Teams) to facilitate remote working and collaboration.	Inadequate resourcing may cause frustration and diminish work efficiency.
**Processes**	**Streamlined & Adaptive Procedures**	Established procedures for notifications and meetings, which expedited responses. Procedures were adapted over time in response to demand, which further streamlined the process and reduced burden.	Procedures need to be purposeful and responsive to reflect dynamic needs, otherwise may be viewed as burdensome and unnecessary.
	**Multidisciplinary Collaboration & Strengthening Relationships**	Multidisciplinary composition of OMT, with well-established roles and responsibilities of its members. Expedient access to expertise and advice from infection control professionals and clinicians. Increased strength of relationship between LHD and RACHs to better prepare for future issues.	Similar to system level integration, roles of members need clear delineation, otherwise their presence may be considered excessive and unnecessary.
	**Open Communication Channels & Teleconferencing**	Open and accessible communication and reporting channels. Establishing a common understanding that communication may extend beyond working hours. Turning on cameras during teleconferencing to improve engagements.	Multiple channels of communication led to conflicting information and high reporting burden. Social isolation facilitated by remote working. Difficulties with administering certain activities remotely (i.e., telehealth, IPC). Volume of communication led to high rates of fatigue, burnout and emotional exhaustion.
	**Provision of Onsite Support & Long-Term Capability Building**	Multimodal and accessible education and advice. Onsite visits be IPC professionals to provide practical education and recommendations specific to the home. Education strategies targeted to ensure sustainability and long-term capacity and capability to manage outbreaks.	This approach is resource-intensive, and therefore must be tailored to prioritise sustainability.
	**Balancing Infection Control & Wellbeing**	Creative methods for mitigating the impact of strict IPC measures.	The balancing of resident wellbeing with IPC measures was not ideal and still needs improvement. Current methods of rectifying the impact of social isolation and visitor limitations are inadequate.

#### Resources and staffing.

Inadequate resourcing within homes was a major structural factor reported to hinder the outbreak response. Over one-third (37.0%) of OMT members identified the supply, or lack thereof, PPE in aged care homes to be a challenge during the initial Delta period where supply was systemically scarce. To address these shortages, WSLHD OMT members provided nursing staff and PPE to RACHs. This was seen as beneficial by participants as it helped to sustain their operations:

They were one of the only ones, of the very few, not the only, one of the very few districts that really actively provided a nursing workforce into the residential aged care homes. (OMTP023)

Interestingly, the supply of PPE and testing kits would also come to elicit secondary problem with regards to resources and infrastructure for RACHs. All RACH participants reported an overcompensation in supplies received from the Commonwealth, which resulted in difficulties for storage and waste management as many homes were not equipped with the storage capacity or infrastructure to handle and dispose of large quantities of product.

…during that time we were having like 30 pallets of PPE turn up completely unannounced and we had to deal with those kind of things like that person. (RACHP001)

The other challenge with infection control was the amount of the waste almost every one hour. So… the rubbish will just pile up outside and then we call them, we call them. They will say we’re coming. We’re coming. We’re coming. But you know they will take at least a couple of days to come. Again, that was a massive. The waste management was the massive problem. (RACHP007)

Resourcing issues also extended to the workforce, whereby outbreaks and furloughing caused substantial staff shortages. Inadequate staff training and knowledge of infection control during the initial outbreak period was also reported to be the source of adverse outbreak management outcomes, through incorrect donning and doffing of PPE, reuse of PPE due to lack of supply, failure to establish appropriate zoning and cohorting procedures, and the aggregation of staff during and outside of work.

At day three or four, we lost about 80% of our staff, you know from 87 staff. We went down to 17 staff around that number and you can… imagine we are in a high care dementia behavioural home. (RACHP006)

In general, aged care homes administered within larger organisations were observed to be better resourced compared to single-site homes, which was considered to improve the likelihood of positive outcomes. This not only applied to the resourcing of PPE and the workforce, but also to the distribution of educational materials, allowing unaffected homes to engage prophylactic measures and be better prepared before an outbreak occurred.

So because we’re a large company, we have lots of educators in Home Office and we do online education and also weekly education and initially all that team came and ran. (RACHP002)

Similar to RACHs, the structural importance of adequate human resources and staffing was also identified within OMTs. During the Delta period, many OMT staff were seconded from their substantive roles to fulfil obligations within the outbreak management response. On the one hand, participants reported that WSLHD was an exemplar jurisdiction due to its provision of staffing, expertise and other material resources to RACHs:

I sat on the State meeting … they thought the way Western Sydney had done it probably was a better approach. Not probably. I think they were hoping that the others would take the same approach … (OMTP021).

I know that the State looks to Western Sydney as an exemplar and took a lot of our documentation and learning to help other local health districts … (OMTP017)

However, while temporarily effective, most OMT members cautioned the strategy to be resource-intensive and ultimately, unsustainable. This was evidenced when most seconded staff returned to their substantive roles at the beginning of the Omicron period and the requirement to train incoming staff became a notable burden.

And the other distress was that after the first initial wave, everyone went back to their substantive positions, and we were left with nobody that was trained and able to manage the subsequent wave. (OMTP003)

… it was an absolute nightmare for them to be staffed appropriately and to maintain infection prevention and control measures. (OMTP027)

### System-level integration

A key structural strength was the integration of the OMT within the wider LHD public health system. The WSLHD OMT response was system-wide and engaged multiple levels and representatives across the LHD, the PHU, and state and federal levels. For OMT members, the scale of the response emphasised the seriousness and urgency of the issue and fostered a strong sense of unity, team effort and a collaborative culture. Similarly for RACH members, the integration was advantageous as it provided clear avenues for receiving advice and connecting with supply chains.

…that was one of the great things with the support we got from the Commonwealth. I say one of them. That’s because that was really challenging and that’s yeah, the Commonwealth relationships and they provided… a great numbers of PPE for us… (RACHP001)

You developed quite good bonds with everyone because you were going through some trying times and… we all just did the best we could… I think everyone was relatively positive despite different times of stress when people were able to complete the tasks or things became more challenging… the overall camaraderie was nice. (OMTP008)

However, some participants questioned whether this level of involvement was excessive and postulated for a more measured approach to resource provision. In particular, as the capacity of RACHs and OMTs increased, the perceived utility of the established reporting and meeting requirements declined. Several participants specifically questioned the purpose of some of the reporting to state and federal authorities:

… they were asking about things that happened last week, like it just seems so irrelevant … this is a waste of my time and I didn’t get anything. (RACHP001)

My reflection is that sometimes Western Sydney stamped resources where maybe they weren’t needed and what is it that you need to do, to work with your residential aged care home, is to be able to kind of assure them that they have what they need … that could be done in … a structured checklist type way. (OMTP023)

### Physical workspaces and digital infrastructure

OMT members identified that consistent access to physical workspaces and relevant digital infrastructures was crucial for facilitating the efficiency of the response, with the lack of access often causing frustration and delays. This critically included access to teleconferencing and collaborative software, which would become the primary point of correspondence and communication between members of the OMT and RACHs. Use of remote working and data management software were regarded as instrumental to the progression of the OMT response in facilitating collaboration despite geo-temporal constraints. However, the initial insufficient availability of computers and screens made leveraging advantageous software difficult, as one participant recalled:

… I was lucky enough that I’d typically always have two screens, and I know some people were stuck with one … and it would be very difficult to manage with one screen. (OMTP020)

### Processes and outcomes

Participants identified the streamlined notification-to-response procedures, the engagement of multidisciplinary partnerships, open communication and the provision of sustainable education and long-term capability building as key strengths of the OMT process.

### Streamlined and adaptive procedures

In general, the outbreak notification and meeting assembly processes were described to be well-structured, streamlined and adaptative. The procedure from outbreak notification to the assembly of the first OMT meeting was established early and included the distribution of meeting invites to relevant members, structured agendas and documentation for the meeting proceedings. These meetings aimed to quickly characterise the nature and severity of the outbreak, identify key environmental characteristics that would affect infection control (e.g., home layout, resources) and respond to the immediate needs of the home.

I think it really benefited them and us being able to work through those outbreak management plans and get those meetings running and have those familiar faces, you know, the homes would be cool to an OMT meeting that has, you know, hospital executives in it, members of the Commonwealth, the Aged Care Safety Commission was sitting there… (OMTP001).

I feel like Western Sydney had… a very nice structured way of running their meetings and they followed that clear structure and that was really beneficial… (OMTP023)

This preparedness facilitated the timeliness of the initial response, but importantly, it also remained flexible to adaptation and adjustment over time. For example, outbreak notifications were initially communicated over-the-phone, before transitioning to email and then eventually to a structured online form that would streamline the process by collecting information that would normally be communicated during the first OMT meeting. Participants reflected that these adaptations reduced the burden of notifications, particularly during the Omicron period when staff shortages were prevalent.

Because of the sheer volume of the outbreaks, we just started doing things like sending text messages to the home to confirm the meeting times rather than calling them personally. We did things like developing Microsoft Teams forms that the homes would fill in, and we’d get a lot of the information that would ordinarily get from the first meeting beforehand, and we’d be a bit more prepared for it. (OMTP003)

### Multidisciplinary collaboration and strengthening relationships

Overall, OMT processes were facilitated by the clear articulation and delineation of roles within the OMT itself, whereby the composition, roles and responsibilities of its members enabled better collaboration, coordination and communication. These were delineated in documentation and disseminated from the outset of the OMT response. Specifically, collaboration was strengthened by the clarity of roles and the multidisciplinary composition of the OMTs as it ensured expedient access to expertise and advice from infection control professionals and clinicians by both OMT and RACH members.

…it was really interesting because it didn’t really matter what your position you held you just got in there and got your hands dirty. (OMTP022)

Some participants noted the friendliness of WSLHD OMT chairs, which provided a *“lovely and human”* element to outbreak management and reinforced professional relationships. The deep collaboration required to manage infection control across dozens of RACHs further strengthened and consolidated inter- and intra-organisational relationships. From early 2022, numerous long duration outbreaks catalysed certain relationships, which one participant outlined:

But we have now at least established relationships between the LHD, PHN, GPs, clinicians and public health and aged care. And so again, that’s great … (OMTP002)

Furthermore, 75% of participants agreed that the OMT process allowed the establishment and development of stronger and more sustained relationships between the homes and the LHD. The experience gained from constant collaboration and practical infection control from successive outbreaks provided learning opportunities for RACHs. The recognition of this relationship is important, as notably, most aged care homes are private entities that are largely overseen federally and do not operate within the jurisdiction of its LHD. Despite this, homes are integrally tied to the public health resources provided by the LHD, in particular through their access to clinical infrastructure like hospitals, ambulances and telehealth.

I think our visit biggest achievements is developing a good rapport and relationship with these aged care homes. Hopefully they’re also gaining some greater infection control, knowledge and practices throughout the time. (OMTP008)

The OMT response further reinforced the connection between aged care homes and local public health structures, which has resulted in the materialisation of regular education webinars, forums and distribution of digital resources and correspondence. Participants reflected on the advantages of these newly formed connections, particularly through their potential to be harnessed to address future public health matters outside of COVID-19 and outbreak management. Participants were hopeful that strengthened connections between organisations of the OMT members could be used to meet other aged care challenges, as illustrated below:

…it also meant that there was less sort of panic around the outbreaks than that we had better relationship, that we went into homes that we’d already had lots of previous contact with, and it definitely made a difference and we’re able to really affirm for the homes that they did a great job previously managing their outbreaks. (OMTP003)

So I think it has built that sort of collaborative environment because not even just for COVID but you know flu, gastro, like lots of different things; if they happen in aged care homes, public health’s involved. So it’s good to sort of feel a bit more connected to them than before … (OMTP007)

### Open communication channels & teleconferencing

Many OMT participants noted that strong and open communication channels were a key strength of the response, facilitated by a common understanding during the relevant period that communication would necessarily and frequently extend well beyond working hours. Immediate and reciprocal accessibility for advice was seen as beneficial, as one participant remarked:

I guess the communication worked because everyone was accessible. So if I needed to escalate, I knew that I would have no problem accessing who I needed to escalate to. And that would be the same when my phone rang - I answered. (OMTP012)

Furthermore, good communication was considered a key driver for collaboration. Meetings held by members outside of the OMT, such as home update webinars hosted by WSLHD staff or PHU internal meetings, were perceived to be important adjuncts which reinforced and supplemented OMT actions. Teleconferencing software undeniably provided the best avenues for communication in the context of a pandemic and the many movement restrictions that were in place during the early period. One participant recounted how the WSLHD policy of turning on cameras during teleconferences made RACHs feel listened to:

… some of the feedback I got from some of the residential aged care home leaders was that other LHDs did not do the same thing and they found it very hard to feel that they were being listened to or connected with when they could never see the face of the people in the meeting … (OMTP027)

Furthermore, the widespread use of common software across OMTs and RACHs allowed for a convenient and reliable infrastructure that better streamlined the response process.

I think the communication… was really good and we actually progressed to having kind of a shared Teams like tracker worksheet… that both teams could access. So again that made things a bit more efficient. (OMTP015)

However, certain aspects of communication contributed to notable inefficiencies. For example, having multiple stakeholders increased the volume of communication, which was reported to have contributed to RACHs and the LHD receiving conflicting advice and outbreak data, respectively. Remote working and teleconferencing also had notable drawbacks. For example, remote working presented challenges to WSLHD OMT staff who lamented the loss of ad-hoc office conversations. Furthermore, services like telehealth and IPC were difficult to administer remotely. As doctors were unable or unwilling to attend RACHs in an outbreak, telehealth was used but was seen as inferior to physical examination, as disdained by one participant:

The challenge is certainly delivering care professionally remotely. … as in that population, telehealth is very substandard. … that was very challenging, not being able to go in and review patients from time to time. (OMTP016)

Furthermore, while the intensity of the response was advantageous initially, many OMT members noted that excessive communication overstepped work-life boundaries and was prioritised at the expense of balancing mental health and wellbeing. As a result, many OMT members reported high rates of burnout and fatigue, ultimately regarding the intensity of the initial response to be unsustainable in the long-term. Several participants worked overtime in addition to their substantive roles as they were temporarily deployed to the OMTs. Some participants recalled having back-to-back OMT meetings late into the night, which contributed to burnout and exhaustion:

… it was really intense, but there were days in a row where all I ate was toast and there were definitely days where I would really challenge the right time to have a shower. I used to hear the phone ringing when it wasn’t ringing. It was like having a new baby and the baby’s not crying but you’re hearing it crying in your head … (OMTP012)

### Provision of onsite support & long-term capability building

The provision of IPC education, training and support to homes was identified to be a highly advantageous component of the OMT response. In particular, onsite visits by IPC professionals to affected homes to provide practical and tailored direction and guidance were considered especially useful. On the one hand, these visits provided IPC professionals with a clearer conception of the layout of homes to better guide risk assessment, zoning, cohorting and other IPC practices. For RACH members and staff, these visits were a rich source of practical knowledge, application and education that necessarily supplemented more general digital IPC resources. As some participants described:

… he was incredible. And he came through and he mapped out our home, told me where all the zones would need to be, what kind of exit points, entry points, what we need to consider for our residents about the zoning. So it was like a real immersive education opportunity for us … (RACHP001)

…that’s where that’s where public health unit came in. They sort of, you know, gave a fresh set of eyes on how to how to zone, how to approach it and what to do just so that we can establish clear guidelines or clear risk assessment for ourselves. (RACHP005)

Participants noted that the multi-modal facets of IPC education helped better equip homes to manage outbreaks. The education frameworks required to improve infection control procedures was often a resource-intensive component of the outbreak response, however, was broadly identified by OMT members and RACH as an effective, useful and sustainable solution. Home staff were quick to embrace the learning opportunities, resources and advice provided by OMTs and used this information to develop and initiate stronger outbreaks processes and procedures, as well as to prevent outbreaks from happening in the first place.

…the capacity within the age care sector particularly has developed and honestly their own capacity to manage their own response in a house to do these things, their own capacity to understand…. They have actually now a good understanding about… what they need to do to balance resident wellbeing with… the challenges of outbreak control. (OMPT006)

Training and education strategies that ensured sustained knowledge retention and application were key to allowing homes to eventually respond to outbreaks with greater independence and less reliance on OMTs. The increased independence had important downstream effects, which saw less dependence on the need for ongoing OMT meetings and resources as the Delta period concluded and the Omicron began. Almost all OMTs reported that the intensity of the response decreased over time and that this was most likely due to the increased capability and growing independence in RACHs. Two corroborating accounts recall this development:

We’ve learned a lot from our first outbreak. We knew exactly what we had to do and what infection control measures had to be put in place to actually control this infection. So we started doing that straight away with our second outbreak … (RACHP006)

… they started to actually tell us what they were doing as opposed to us saying you should be doing this... (OMPT006)

### Balancing infection control and resident wellbeing

All OMT members described the challenges of balancing the holistic wellbeing of RACH residents with the strict demands of infection control. In particular, measures such as isolation and visitor restrictions were regarded to have a detrimental impact on the psychological wellbeing of residents, as well as their families. Participants lamented the use of severe isolation measures, believing the adverse consequences of such measures on residents’ health did not justify the lowered risks to infection and disease transmission.

I think there’s a very ethical dilemma, and I’m still not sure what the right answer is, of the balance between the public health and humane care of people who are dying and the grieving of the relatives. Some of these old, frail people who died without seeing their relatives; it must have been terrible for both sides … (OMTP013)

Many OMT members advocated for the implementation of strategies to ensure resident wellbeing such as one-on-one, in-room physical therapy and diversional activities. However, some criticised the inadequacy of these expedients, such as those which attempted to address social isolation:

… we put in place telephones and iPads and digital things like that, but it’s not the same and particularly if you’re an older person, it’s sometimes, it’s confusing as well. So I think that had a big emotional impact for people. (OMTP019)

Given these shortcomings, there was a desire for a more nuanced approach to outbreak management to have been implemented earlier so broader outcomes could have been considered. These outcomes highlight that, in comparison to acute care settings, current understanding and practice for infection control in residential aged care is still in its infancy – and importantly, that recommendations from acute care must be applied with careful consideration. As one participant reflected:

I think a bit more generic and less prescriptive … I think embedding an understanding of the importance of non-infectious diseases, resident welfare … so you know physical activity, visiting family members; you know the psychological effect of isolation … I think in future, it would be good to incorporate that explicitly. (OMTP015)

## Discussion

The study used the Donabedian framework to explore the structures, processes and outcomes of the OMT response for the prevention and management of COVID-19 outbreaks in Western Sydney RACHs [37]. This was achieved using a mixed methods design, which collated outbreak data from several public health sources which were then supported by findings from in-depth, semi-structured interviews with key actors involved in the response. To our knowledge, this is the first reported Donabedian health evaluation of such an outbreak management response to date.

Several structural factors were identified to contribute to the efficacy of the OMT response, and many of these factors concurred with the broader literature on outbreak management in RACHs. In general, homes that were larger in size (i.e., greater bed numbers) were associated with higher rates of COVID-19. This observation was corroborated by members of OMTs and RACHs, who recognised that larger homes, as well as those with complex layouts and shared rooms, often posed as barriers to establishing effective cohorting and infection control measures. These findings are consistent with existing research internationally and in Australia, which have similarly isolated home size and design as key contributors to higher outbreak risk [32,43–51]. Furthermore, homes that had dementia residents or dementia-specific units were also associated with worse COVID-19 outcomes. Dementia is a well-established risk factor for adverse infection outcomes, both as an independent physiological risk factor for residents’ health, as well as an infection control risk factor due to its association with wandering behaviours, and greater non-compliance and resistance to key infection control measures [14,52–54].

Availability and accessibility to resources and staffing was also considered an important structural factor, and one that was importantly augmented by the integration of OMTs and RACHs within wider systems and networks. Participants identified that the intensity and whole-of-system scale of the response emphasised its urgency and importance, and thereby empowered a sense of purpose and camaraderie amongst its members. Studies in the US and Europe have similarly found that having a coordinated public health approach, and that having collaborative relationships with hospitals, local public health official and infection control experts, facilitated better health and COVID-19 outcomes for aged care homes [55–57]. For example, a qualitative study completed with German aged care home managers identified collaboration with external service providers, in addition to high motivation and a sense of unity, as a key supporting factor for improving the home’s response in the face of an outbreak [31]. In a similar vein, homes belonging to broader organisations that administered multiple homes had lower rates of COVID-19 compared to single standing homes. These findings are congruent with a large US study which found that ‘chain’ homes were less likely to have at least one COVID-19 case [45]. Homes administered by larger organisations also likely have greater capacity to centralise resources, such as PPE, testing, education and staff, across their sites, resulting in better resilience and capability to manage or prevent outbreaks.

In general, the integration of the WSLHD OMT response within wider public health structures allowed for the prompt assembly of support chains and the expedient supply of education and IPC resources, which were greatly beneficial to homes and outbreak outcomes. Indeed, having adequate resourcing and staffing within both RACHs and OMTs was reported to be crucial for facilitating the response, and the lack thereof exacerbated existing deficiencies in knowledge of COVID-19, infection control non-compliance and increased burden on the existing workforce. For homes, inadequate staffing levels, dependence on agency staff and the lack of supply and access to IPC resources and education have all been previously linked with greater likelihood of COVID-19 outbreaks [30,31,47,51,57–60]. Similarly, for OMT members, the magnitude of the initial response resulted in considerable exhaustion, and this was further compounded when many staff returned to their substantive positions following the Delta period. These outcomes support recommendations for more systematic resource provision to limit strain on staff and resources, as seen in other jurisdictions [30,31,61].

The processes that facilitated the OMT response included multidisciplinary collaboration and open communication, which in-tandem streamlined outbreak notification-to-response procedures. Outbreak management is an exercise of extensive collaboration [28,29,34], and OMT members valued the development and utilisation of relationships during the response process. Establishing infrastructure for work and communication was also critical for fostering collaboration and streamlining the OMT response, as communication efficiency was imperative for ensuring that the response was timely and informed [44,60]. Ultimately, OMT collaboration depended on two-way communication [44] and participants reported that substandard communication channels often led to inefficient practices. Additionally, the quantity of communication also became an issue long-term where fatigue and frustration developed as a result of excessive reporting and administrative burden. The sentiments towards unclear communication channels and high reporting burden are mirrored with findings internationally [62–64], and indicate the need for future processes to be more adaptive, purposeful and most importantly, sustainable for both OMT and RACH staff.

Another key process identified to facilitate the OMT response was the provision of onsite IPC education and support. Participants perceived these experiences to have improved their confidence and capabilities in managing COVID-19 and other infectious diseases. Similar findings have been observed for the effect of interactive educational programs for increasing knowledge of key IPC principles [65]. Other studies completed in Australia have also indicated that onsite and practice education provided greater engagement, understanding and compliance [34,35,66,67]. Furthermore, activities such as the provision of onsite IPC support and education, delivery of online education content and forums (e.g., webinars) and the linkage with supply chains for resources like PPE and testing kits were all included as part of an interventional study that successfully reduced COVID-19 prevalence in Massachusetts homes in the US [68]. Similar strategies have also been reported in Canada, which implemented multidisciplinary IPC teams to provide onsite IPC support through education, training and engagement both as a dedicated intervention [29], and as part of a broader outbreak bundle [28]. Overall, these studies have reported positive COVID-19 outcomes post-intervention, including ongoing partnerships with included homes and high self-reported efficacy and capability to manage further outbreaks from staff [29], as well as reduced incidence of COVID-19 in the home amongst both residents and staff [28].

Finally, the tension between infection control and maintaining holistic resident wellbeing has been abundantly described in the literature [28,29,69–71], and also emerged as a key critique of the OMT process in the present study. The health and wellbeing of residents were severely impacted during the pandemic, not only as a result of COVID-19 transmission, but also as a consequence of stringent infection control measures, such as isolation, cohorting, the restriction of movement and the limitation of visitations from family members and friends. These restrictions had profound impact on residents’ emotional and social wellbeing as they limited resident’s opportunities to have frequent and meaningful social interactions, inhibited their ability to engage in leisure activities and caused significant disruption to their regular routines. Studies have reported the independent impact of IPC measures on both the physical and mental health of residents and their families, typically manifesting as signs of physical deterioration, such as reduced oral intake, weight loss, increased pain, reduced activities of daily living, depression and reduced cognitive function [72, 73]. These effects were keenly observed and felt by both OMT and RACH staff, many of whom were acutely aware of the need to balance infection control measures with ensuring residents’ social and emotional wellbeing, and frequently sought pragmatic ways to attain it [69]. These findings highlight a pressing deficit in the practice of infection control and the need to ensure future standards advocate for a more holistic approach to IPC in residential aged care.

Overall, our findings indicate that the OMT response in WSLHD was generally well-received by the RACH community and considered to be a useful and beneficial endeavour that led to greater infection control capacity in facilities. The clear articulation of the purpose and governance of the OMT, including a clear delineation of its membership, tasks and roles within the wider public health response [22,26], from the start of the pandemic response was critical to its success and implementation. In Australia, RACHs are overseen by the federal government, whereas most public hospitals and health services are the responsibility of state and local jurisdictions. Consequently, the COVID-19 response for RACHs was characterised by a complex coordination of multiple organisations across all levels of local, state and federal government. In this context, OMTs became a key point of operational, logistical and communications support; ran by local health networks that were best positioned to provide targeted, on-the-ground assistance and redirect federal resources to where it was most needed.

There are some limitations to this study. Firstly, there was limited participation in RACH managers compared to LHD staff. Secondly, this is a descriptive study of one specific jurisdiction and its associated RACHs, and the results are not directly comparable to other settings. Differences between outbreak outcomes across the Delta and Omicron periods need to be contextualised within the broader scope of state-wide and national public health measures, which importantly includes the rollout of the vaccine and the easing of restrictions. Furthermore, to better assess the impact of the WSLHD OMT response, these data need to be compared to outbreak outcomes across other LHDs in NSW. Strengths of this study include the availability of data from all WSLHD RACH outbreaks for analysis and with a mixed-methods design, the rich descriptions of OMT members’ experiences gave depth to these outbreak statistics.

## Conclusion

The vulnerability of RACH residents to COVID-19 infections requires effective outbreak management, and the OMT is core to this response. This study’s evaluation of the structures, processes and outcomes of the OMT response contributes to the literature of RACH COVID-19 outbreak management which has not been subject to systematic and sustained research in Australia. Overall, the key facilitators to the OMT response included the clear delineation of OMT roles and procedures, multidisciplinary and inter-organisational collaboration, strong communication channels, judicious deployment of resources and the provision of sustainable education to ensure long-term capability and preparedness. The lessons learned from this period may be applied to future outbreaks and contribute to positive resident outcomes and the sustainability of any response.
